# Beta activity in the premotor cortex is increased during stabilized as compared to normal walking

**DOI:** 10.3389/fnhum.2015.00593

**Published:** 2015-10-27

**Authors:** Sjoerd M. Bruijn, Jaap H. Van Dieën, Andreas Daffertshofer

**Affiliations:** ^1^Department of Human Movement Sciences, MOVE Research Institute Amsterdam, VU University AmsterdamAmsterdam, Netherlands; ^2^Department of Orthopaedic Surgery, First Affiliated Hospital, Fujian Medical UniversityFuzhou, China

**Keywords:** gait stability, EEG, beta power, premotor cortex, motor control

## Abstract

Walking on two legs is inherently unstable. Still, we humans perform remarkable well at it, mostly without falling. To gain more understanding of the role of the brain in controlling gait stability we measured brain activity using electro-encephalography (EEG) during stabilized and normal walking. Subjects walked on a treadmill in two conditions, each lasting 10 min; normal, and while being laterally stabilized by elastic cords. Kinematics of trunk and feet, electro-myography (EMG) of neck muscles, as well as 64-channel EEG were recorded. To assess gait stability the local divergence exponent, step width, and trunk range of motion were calculated from the kinematic data. We used independent component (IC) analysis to remove movement, EMG, and eyeblink artifacts from the EEG, after which dynamic imaging of coherent sources beamformers were determined to identify cortical sources that showed a significant difference between conditions. Stabilized walking led to a significant increase in gait stability, i.e., lower local divergence exponents. Beamforming analysis of the beta band activity revealed significant sources in bilateral pre-motor cortices. Projection of sensor data on these sources showed a significant difference only in the left premotor area, with higher beta power during stabilized walking, specifically around push-off, although only significant around contralateral push-off. It appears that even during steady gait the cortex is involved in the control of stability.

## Introduction

That humans excel in intellectual capacities is common sense, but we also outperform large parts of the animal kingdom with our motor repertoire. As such, we perform bipedal gait with remarkable ease (Alexander, 2004) although bipedal gait is inherently unstable and requires continuous and highly sophisticated adjustments (Kuo, 1999), which causes both toddlers and elderly to struggle.

Gait stability comprises at least three aspects (Bruijn et al., 2013): proactive, reactive, and steady-state gait stability. According to dual-task studies, the latter already involves cortical control (Woollacott and Shumway-Cook, 2002), at least to some extent. Bruijn et al. (2014) sought to associate neuro-anatomy, quantified as DTI-based structural integrity, with different measures of gait stability. In elderly, they found significant correlations between the structural integrity of the left anterior-thalamic radiation and cortico-spinal tract and the medio-lateral margin of safety of the body's center-of-mass relative to the lateral border of the foot, which were age-independent. Arguably, balance in the medio-lateral direction is controlled during gait by higher cortical centers, first and foremost in the left hemisphere (Sipp et al., 2013; Bruijn et al., 2014).

Functional magnetic resonance imaging (fMRI) or related techniques have been frequently employed to unravel neural correlates of gait control (Fukuyama et al., 1997; Jahn et al., 2004, 2008; Hanakawa, 2006; Bakker et al., 2007; Wang et al., 2008; Snijders et al., 2011). Unfortunately, fMRI acquisition does not allow subjects to move, let alone to walk, and studies remain restricted to imagined gait. Near infrared spectroscopy (NIRS), by contrast, does allow for assessing cortical activity while walking (Harada et al., 2009). However, NIRS has a fairly limited spatial and temporal resolution, which limits detailed search for neural correlates of dynamic gait stability. Despite their shortcomings, both fMRI and NIRS studies suggest that (imagined) gait is accompanied by activity in an extended network of brain areas, including subcortical (Jahn et al., 2004, 2008) and cortical structures (Fukuyama et al., 1997; Miyai et al., 2001; Dobkin et al., 2004; Hanakawa, 2006; Bakker et al., 2007; Wang et al., 2008; Harada et al., 2009; Snijders et al., 2011).

Overall, the roles that brain areas and their connectivity play in controlling gait stability remains largely unclear. Identifying such a role mandates measurement of brain activity at high temporal resolution, and concurrent assessment of gait stability. In order to associate the corresponding process(es), the demands for control of gait stability should be altered (either by stabilizing gait, or by destabilizing gait), so that changes in brain activity can be related to changes in control of gait stability.

Recently, high-density electro-encephalography (EEG) was presented as an alternative method to study cortical function during gait (Gwin et al., 2010, 2011; De Sanctis et al., 2012, 2014; Debener et al., 2012; Severens et al., 2012; Wagner et al., 2012, 2014; Sipp et al., 2013; Seeber et al., 2014; Malcolm et al., 2015). This application of EEG is remarkable, as traditionally, movement artifacts had been considered a prime confounder. Among others Gwin et al. (2010) and Severens et al. (2012) showed how to overcome this limitation, by exploiting recent advances in data processing. Of particular interest for the role of the cortex in stabilizing human gait is the study of Sipp et al. (2013), which revealed that walking on a balance beam may lead to significantly reduced power in the beta frequency band in left and right sensory motor cortex, as well as an increase in theta power in or near anterior cingulate, anterior parietal, superior dorsolateral-prefrontal, and medial sensorimotor cortex. This is an indication of the involvement of these areas in the cortical control of gait stability. However, the study of Sipp et al. (2013) was performed at very slow walking speeds, potentially limiting generalization to real-life walking. Recent studies (Castermans et al., 2014; Kline et al., 2015) suggest that lower frequency bands may be heavily contaminated with movement artifacts, and it may be that the extra movement caused by walking on a balance beam led to the reported increase in theta activity. On the other hand, theta band activity has also been shown to be involved in standing balance (Hülsdünker et al., 2015).

In the current study, we investigated the role of cortical activity in gait stability by measuring EEG during gait, while simultaneously measuring kinematics to assess gait stability. To manipulate stability demands, we opted to stabilize subjects by means of elastic bands (Donelan et al., 2004; Ijmker et al., 2013). In this so-called stabilized walking condition, control of medio-lateral motion is aided by the force field generated by the springs (Kuo, 1999; Bauby and Kuo, 2000; O'Connor and Kuo, 2009).

We hypothesized that stabilized walking would lead to an increase in beta activity in left and right sensorimotor areas.

We further expected that modulations in beta power over the gait cycle would reflect phase dependent variations of stability over the gait cycle (Ihlen et al., 2012). For instance, it has been shown that the weight transfer phase (i.e., the phase between heelstrike of one leg and toe-off of the other leg) is critical for gait stability (Ihlen et al., 2012). Thus, during normal gait, we expected decreased beta activity during unstable phases (transfer phases, mid-swing), and increased activity during more stable phases. Moreover, we expect that phases that are specifically important for gait stability in the mediolateral plane would show the largest differences in beta power between conditions.

## Methods

### Subjects

Ten healthy subjects [7 males, age 31.4 ± 6.6 years (mean ± sd), length 1.79 ± 0.09 m, weight 67.1 ± 9.6 kg] recruited by word of mouth within the Faculty of Human Movement Sciences participated in the experiment. All subjects signed an informed consent form before participation. The protocol had been approved by the ethics committee of the Faculty of Human Movement Sciences, VU University Amsterdam, Amsterdam, The Netherlands.

### Subject preparation

Subjects wore a 64-electrode EEG cap (TMSi, Twente, The Netherlands). Electrode-skin contact was improved with gel (SonoGel, Bad Camberg, Germany) to guarantee impedance below 20 kOhm. EEG montage agreed with the 10–20 standard. We used an average common reference. In order to aid *post-hoc* removal of muscle activity in the EEG, two pairs of bipolar EMG Ag/AgCl electrodes (Ambu blue sensor N, Ambu, Ballerup, Denmark) were placed bilaterally on the M. Trapezius pars descendens. EEG and EMG were recorded using a TMSI Refa 64-channel amplifier (TMSI, Twente, The Netherlands) and digitized at a rate of 2048 samples/s.

To ensure safety, subjects were outfitted with a safety harness. To measure 3D trunk kinematics, cluster-markers containing three infrared light-emitting diodes were attached to the thorax (at the level of Th6) and both feet around the heels. Kinematic data were recorded using an Optotrak 3020 system (Northern Digital, Waterloo, On, Canada), which was placed behind the treadmill, and signals were digitized at a rate of 100 samples/s.

To test our data cleaning procedures, we included two conditions during which we applied electrical stimulation of the medial nerve (while sitting, and while walking, see procedures). Appropriate data cleaning should not lead to reduction in amplitude of the SEPs during both conditions. Hence, we mounted stimulation electrodes over the left medial nerve (at the level of the wrist). The stimulus duration was 200 μs and stimulus rate varied randomly between 2 and 3 pulses/s; intensity of the stimulation was fixed to be just above perception threshold determined prior to recording.

The entire experiment was performed on an instrumented split-belt treadmill (Forcelink, Culemborg, The Netherlands), with which ground reaction forces were recorded and digitized at a rate of 1024 samples/s. These ground reaction forces were used to calculate the center of pressure (CoP) position, from which gait events were extracted (see below).

Subjects were instructed to move their head as little as possible during the entire experiment, and to look straight ahead to a fixation cross that was placed about 5 m in front of them at eye-level.

### Procedures

The experiment consisted of five conditions; two baseline conditions, and three treadmill conditions.

The two baseline conditions consisted of (1) sitting, eyes open for 1 min, and (2) sitting, eyes open, while the subject received a total of 1000 stimuli to the medial nerve (in about 7 min).

During each of the three treadmill conditions, subjects walked at 1 m/s for 10 min.

**Figure 1:**
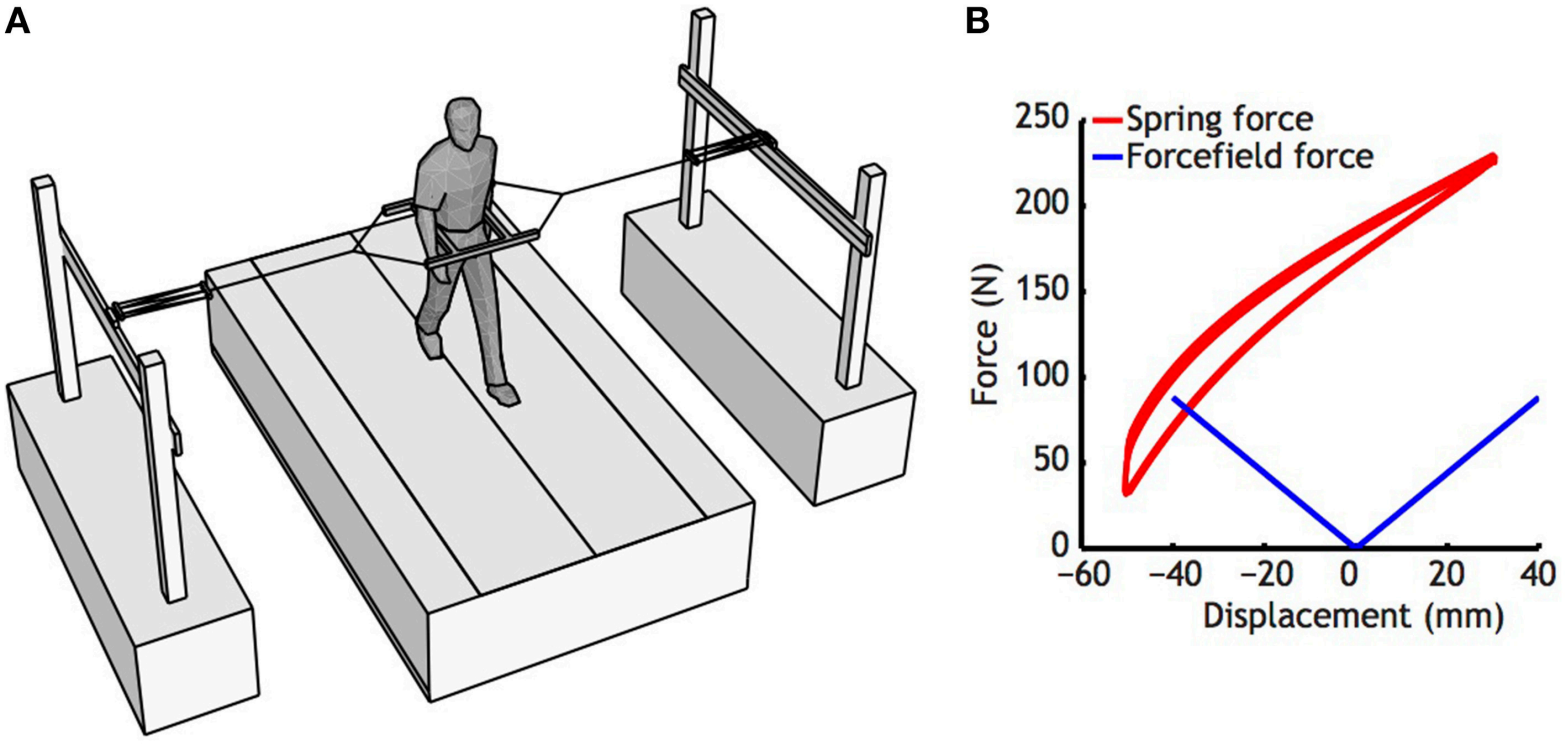
**(A)** Set-up used to stabilize the subjects. Note that subjects were only restricted in medio-lateral direction, as carts could freely move on rails in anterio-posterior direction, and vertical movements have little effect on the length of the springs. **(B)** Characteristics of the springs used (Red lines), and the force field generated by the spring used. Springs were pretensioned, so that in the initial position, the springs left and right exerted about 150 N. For estimation of the force field, a regression was fitted to the whole displacement curve of the spring, ignoring slight differences between lengthening and shortening that occurred as a consequence of damping of the spring.

In the first treadmill condition, subjects walked normally without further manipulation. In the second treadmill condition, subjects were outfitted with a custom-made frame around the pelvis. This frame was tethered to the outside world via two elastic cords, attached to two carts that allowed fore-aft movement (see Figure 1, Donelan et al., 2004; Ijmker et al., 2013). This set-up stabilized medio-lateral movement without constraining the anterio-posterior direction. In the third treadmill condition, subjects walked while receiving the same stimulation they had received while sitting.

### Data analysis

#### Gait parameters

Heelstrikes and toe-offs were automatically detected from reversal points in the CoP following Roerdink et al. (2008). In brief, a heel strike was defined as the point at which the CoP starts to move forward, toe-off was the point at which the CoP starts to move backward, and left and right could be determined by the CoP position in the ML-direction. The extracted heel strikes and toe offs were verified, and whenever missed by the algorithm, reversal points were added manually, and false positive reversal points were deleted. We used the CoP data (instead of kinematics) for this aim because it was sampled at a higher rate, and thus, was more suitable to temporally align with the EEG. Standard kinematic parameters (step width, step length) were determined from the AP and ML distances between the foot markers at heel strike times. To quantify changes in dynamic stability due to stabilization we also calculated the range of medio-lateral trunk movement, as well as the local divergence exponent of trunk movement. The latter was calculated from the medio-lateral trunk cluster velocity (Rosenstein et al., 1993; Rispens et al., 2014). An equal number of strides was used for both conditions (*n* = 300), and these 300 strides were time normalized to 300 × 100 samples (retaining temporal variability). Next, state-spaces were reconstructed with embedding dimension five, and a time delay of 10 samples. The local divergence exponent was determined from the divergence curve from 0 to 0.5 stride, expressed as log(divergence)/stride (Stenum et al., 2014).

#### EEG

##### Data cleaning

All EEG data processing was performed using the Fieldtrip toolbox (http://fieldtrip.fcdonders.nl/, Oostenveld et al., 2011). EEG and EMG data were high-pass (2nd order bi-directional 3 Hz Butterworth filter), and band-stop filtered (1st order bi-directional at 50, 100, 150, and 250 Hz, to remove line-noise) and down-sampled to 512 samples/s. EMG data were additionally high-pass filtered at 20 Hz (1st order bi-directional Butterworth). After filtering, data were visually inspected, and episodes and channels with large artifacts were discarded (0–3 channels, on average 1.1 channel removed per subject).

Subsequently, EEG and EMG data for each condition of each subject were subjected to independent component (IC) analysis (Bell and Sejnowski, 1995; Gwin et al., 2010, 2011). The resulting ICs were classified as (1) muscle artifacts when components had a mean power in the 50–100 Hz frequency band larger than that in the beta and/or alpha bands, and components' loading mainly in EMG channels; (2) eye-blink artifacts when the median frequency was low (below 3 Hz) and the topological map corresponded to eye components (one component per trial); (3) movement artifacts when components had a frequency spectrum at harmonics of the movement (Castermans et al., 2014; Kline et al., 2015); or (4) EEG components (see Table 1 for overview). Only the EEG components were retained, and projected back onto the sensors. With these “artifact-free” signals we interpolated the previously discarded improper channels using the average of their close-by neighbors weighted by distance.

**Table 1:** **Amount of independent components removed because they were eye-blink, muscle, or movement artifacts**.

	**Sit**	**Sit+Sepp**	**Walking+Sepp**	**Walking**	**Stabilized walking**
Eyeblink components	1.0 [1.0–1.0]	1.0 [1.0–1.0]	1.1[1.0–2.0]	1.0 [1.0–1.0]	1.0 [1.0–1.0]
Muscle components	9.5 [2.0–22.0]	10.0 [0.0–25.0]	11.5 [2.0–24.0]	12.5[3.0–24.0]	9.7 [2.0–26.0]
Movement components	0.0 [0.0–0.0]	0.0 [0.0–0.0]	5.7 [1.0–13.0]	5.6 [0.0–10.0]	7.9 [1.0–13.0]
Components remained	52.4 [40.0–60.0]	51.9 [37.0–62.0]	44.6 [30.0–59.0]	43.8 [33.0–58.0]	44.3[30.0–58.0]

*Values represent mean and [range]*.

##### Effects of cleaning data

To assess the effects of data cleaning, we compared the SEP conditions (during both sitting and walking) in both cleaned and un-cleaned data using a conventional event-related approach (note that for this test the data were not down-sampled). We used a baseline of 10 ms, and plotted scalp maps for the response from 35 to 45 ms after stimulation (P40, Desmedt et al., 1983). The average response of C4 for this time-period was also extracted and used to determine whether condition (walking or sitting) or cleaning significantly affected the evoked response.

##### Spectral analysis at sensor level

We performed a spectral analysis at sensor level to identify a frequency band that showed differences between conditions, and would thus be a proper target for beamforming. Hilbert amplitudes at frequency bands from 5 to 40 Hz (steps of 1 Hz, filter bandwidth of 2 Hz) were determined. These time-frequency representations of the data had the same length as the original data which allowed for using conventional shape preserving splines to time-normalizing them to gait events. From left heel contact → right heel contact was (re-) sampled to 256 samples (i.e., half a second, close to stride time), and from, right heel strike → left heel strike was sampled to 256 samples. Subsequently, the average over gait cycles [normal walking 81–457 (mean 352) cycles, stabilized walking 83–450 (mean 364 cycles)] was taken yielding mean power spectra of a gait cycle. Results were further normalized by the sum of the Hilbert amplitude over time and frequency. That is, power spectra were determined as mean over time of the normalized time-frequency representations of Hilbert amplitudes^1^.

##### EEG source analysis

Given the results of sensor level analysis, we performed dynamic imaging of coherent sources (DICS; Gross et al., 2001) beamforming in the lower beta band (18 Hz, with a bandwidth of 2 Hz), creating a common filter based on the sensor-level coherences of combined walking and sitting^2^ data for every subject. We then mapped sensor-level power from these conditions via this spatial filter onto source level.

We used a four-element boundary-element forward model of a template MRI (Oostenveld et al., 2003) with a 5 mm spaced source grid. For some subjects (*n* = 6), the source analysis yielded activity in unlikely sources (e.g., related to muscle locations) because of which several posterior and/or temporal channels had to be excluded from analysis (overall, 0–7, mean 2.3 channels per subject were removed, based on visual inspection of the power spectra). The estimates of source power obtained for both (sitting and walking) conditions were compared using a paired *t*-test clustering procedure (Maris and Oostenveld, 2007) to identify sources with significant changes in power between conditions.

We searched for the highest *t*-value within these clusters and considered this the primary source. The highest remaining *t*-value that was not closer than 4 cm from the primary source was determined and considered the secondary source. This 4 cm was based on the fact that our source localization was performed without individual registration of electrode positions, and without co-registering individual MRIs, which let us expect a fair amount of uncertainty in the location of anatomical sources. We estimated that the error might be in the order of 2 cm.

Next, we projected sensor data to source space. For each subject, and source, we looked for the voxel that showed maximum power difference between the two conditions (sitting vs. walking) and was within a distance of 2 cm from the (group-defined) peak-*t*-value of that source. The DICS filters of these locations were used to project the sensor data of normal and stabilized walking to the sources.

##### Spectral analysis of source activity

On the extracted source level activity, we performed a time-frequency analysis using the same method as previously applied for the sensor level analysis.

### Statistical analysis

Differences in behavioral measures (stride time, stride width, trunk excursion, local divergence exponent) were tested using paired *t*-tests. Differences in SEP amplitude were calculated using a 2 (condition, walk vs. sit) by 2 (cleaning, uncleaned vs. cleaned) repeated measures ANOVA. Difference in source level-power spectra between conditions were tested using a wavelet-based functional ANOVA (McKay et al., 2013). In short, the normalized power spectra were subjected to a wavelet transform (3rd order Coiflet wavelet, with periodic extension), and ANOVAs were performed on the individual wavelet coefficients. To correct for multiple comparisons, we used a Scheffe *post-hoc* test. Wavelet coefficients that were significantly different between conditions were projected back to obtain the spectral difference. Whenever a significant difference in the power spectrum between conditions was found, the time-evolution of power over the gait cycle was tested between conditions using a similar wavelet based functional ANOVA. The Matlab statistics toolbox was used for all statistical analysis; *p* < 0.05 was considered significant.

## Results

### Effects of external stabilization on gait parameters

**Figure 2:**
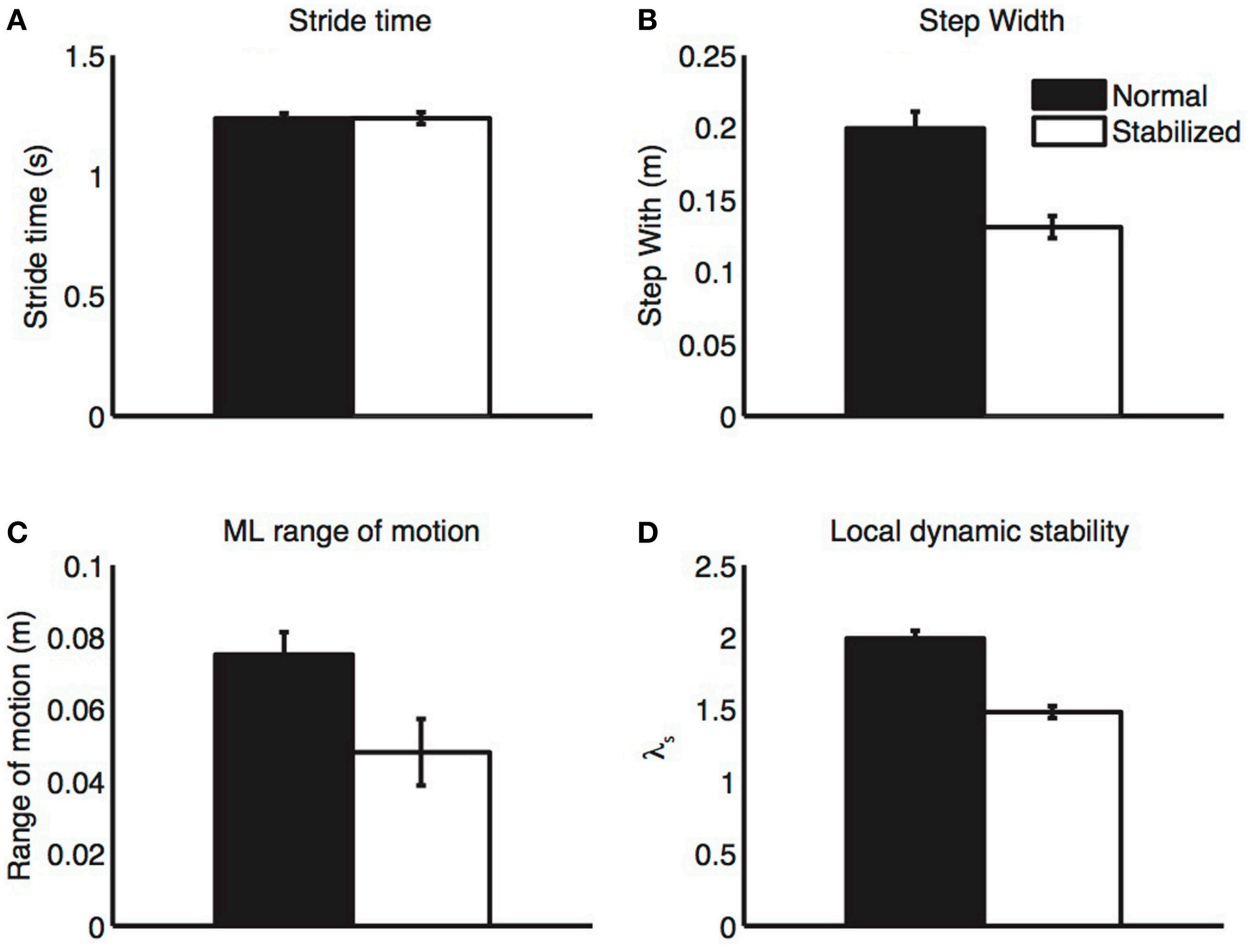
**Gait parameters**. During stabilized walking, subjects walked with similar step length **(A)**, but had clearly reduced step width **(B)**, medio-lateral trunk excursion **(C)**, and local divergence exponents **(D)**, all indicative of increased stability. Bars represent mean values, error bars represent standard errors.

All gait parameters are displayed in Figure 2. Stabilized walking did not change stride time, but led to a significant decrease in step width and trunk excursion and a significant increase in stability (i.e., lower λ_*s*_).

### SEP—effects of cleaning and gait

**Figure 3:**
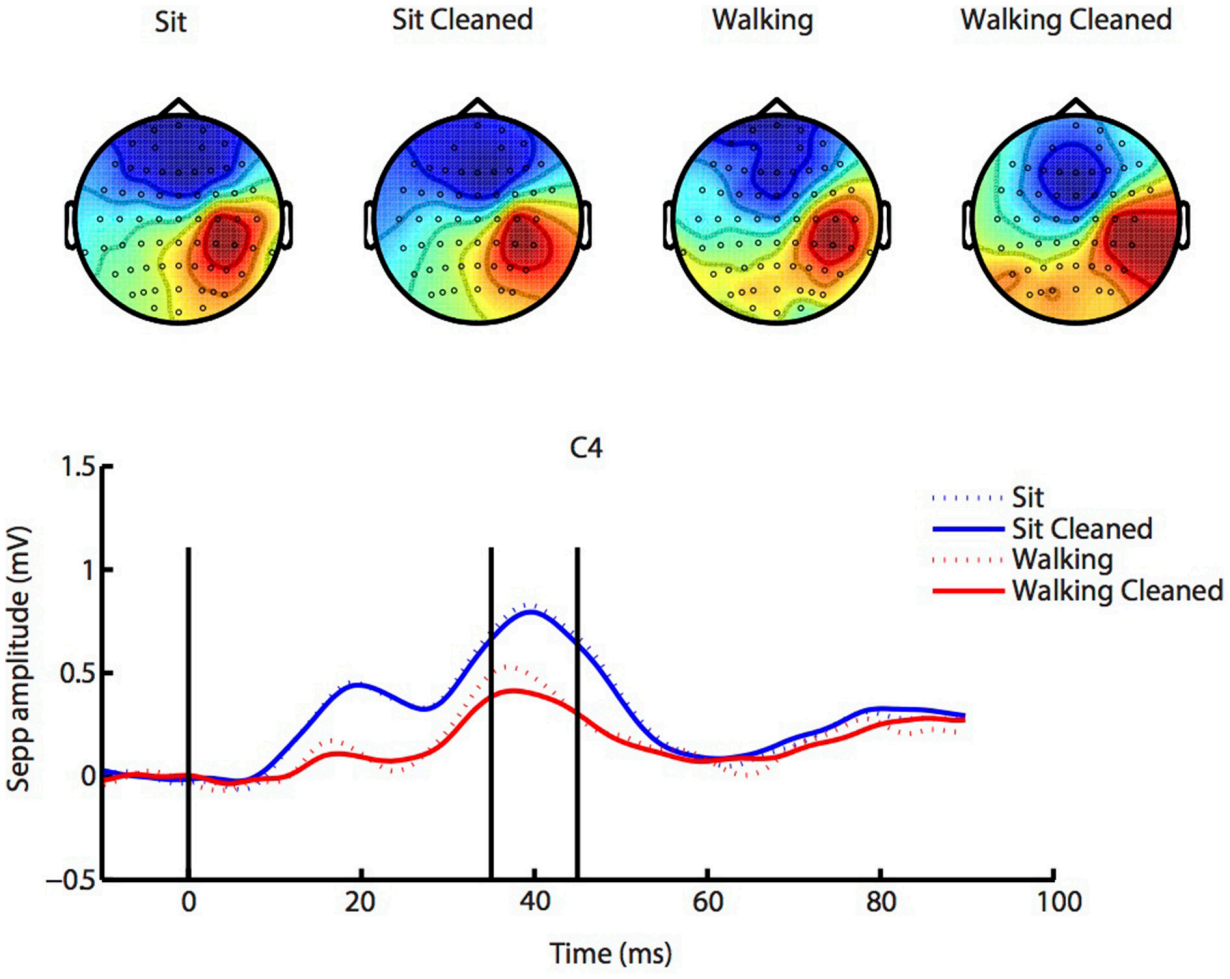
**SEP analysis**. Similar SEP responses and accompanying scalp maps (from 35 to 45 ms post stimulus, indicated by vertical lines) were present during both sitting (blue lines) and walking (red lines), and our ICA cleaning procedure changed SEP amplitude only little (solid lines; cleaned), while clearly improving the scalp map. See Supplementary Figures 1–4 for source localization of SEP activity.

The results for the SEP analysis are depicted in Figure 3. Scalp maps largely agreed between sitting and walking conditions, although stimulation during walking elicited somewhat lower responses^3^. Data processing led to improvements in the quality of the scalp maps almost without changing amplitude, which suggests the validity of our data preprocessing. There were no significant effects of either condition (walking vs. sitting, *p* = 0.09), cleaning (*p* = 0.22) or interaction of these factors (*p* = 0.38).

### Effects of external stabilization on brain activity

#### Sensor level analysis

**Figure 4:**
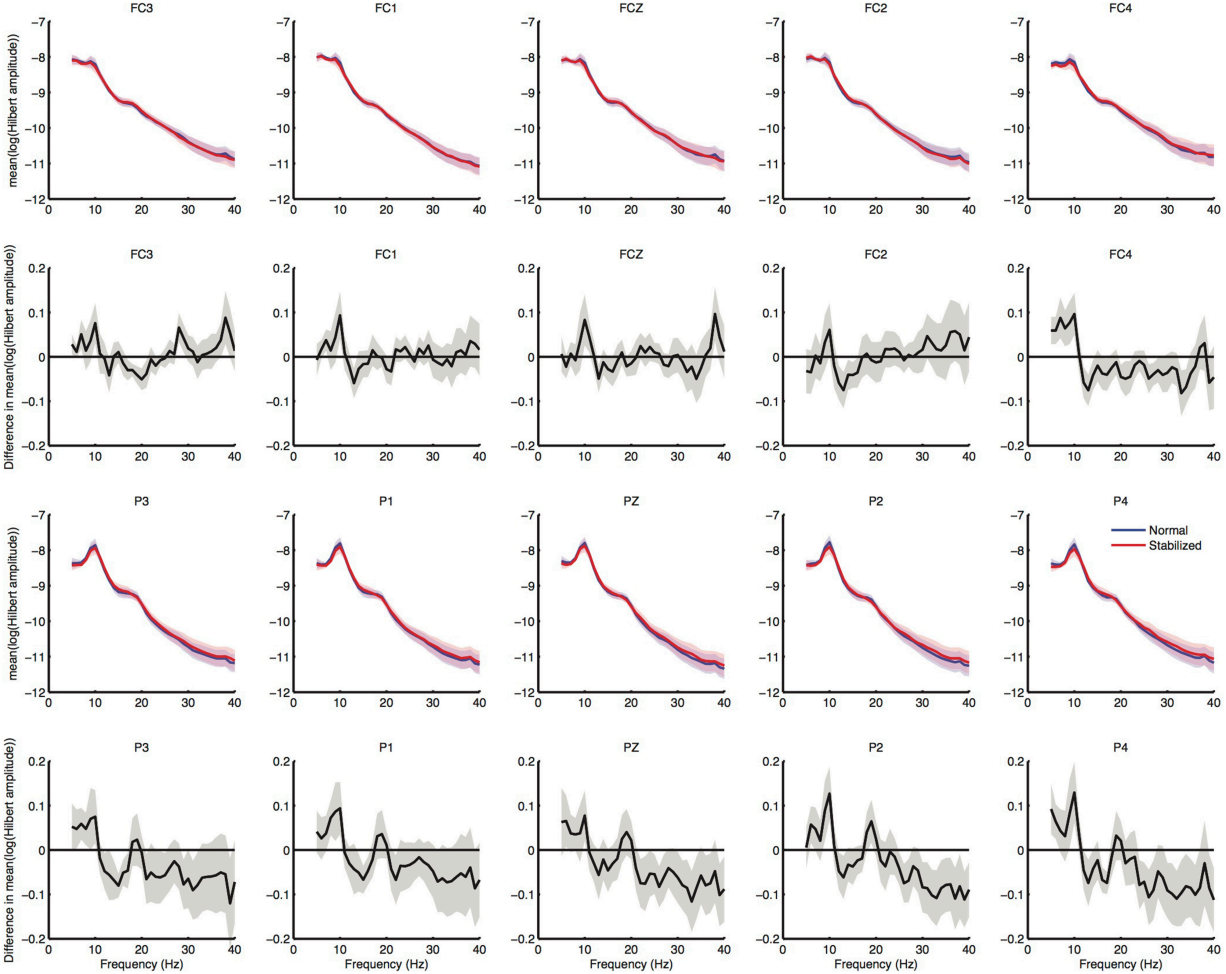
**Results of sensor level spectral analysis**. First row shows normalized power spectra of electrodes over the premotor area, second row shows difference in power spectra between conditions, in which values below zero represent higher power in the stabilized condition. Third and fourth row shows spectra over posterior electrodes for comparison. Shaded regions represent standard errors.

**Figure 5:**
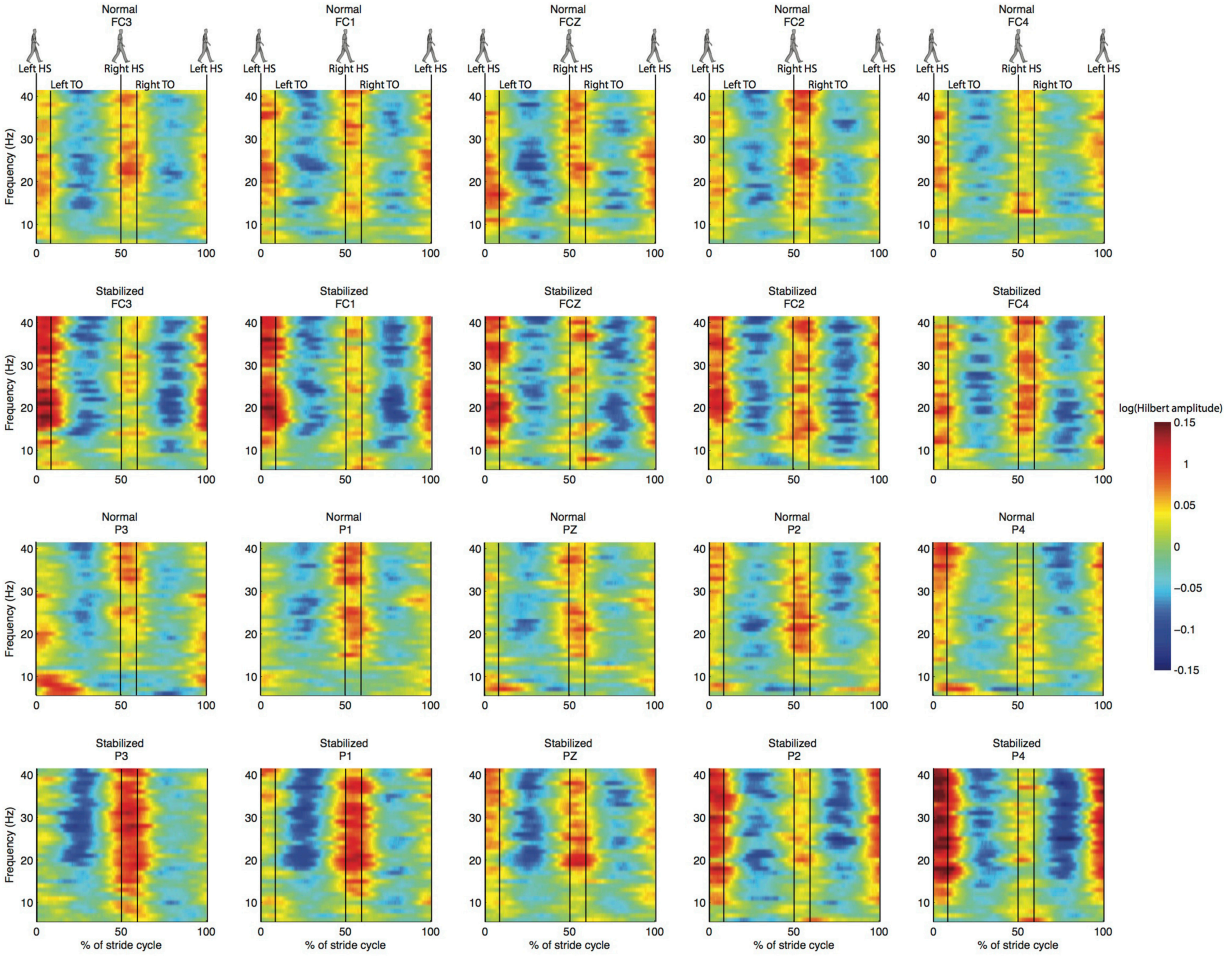
**Normalized ERSP for the same electrodes as in Figure 4**. First and third row are for normal walking, second and fourth for stabilized walking.

**Figure 6:**
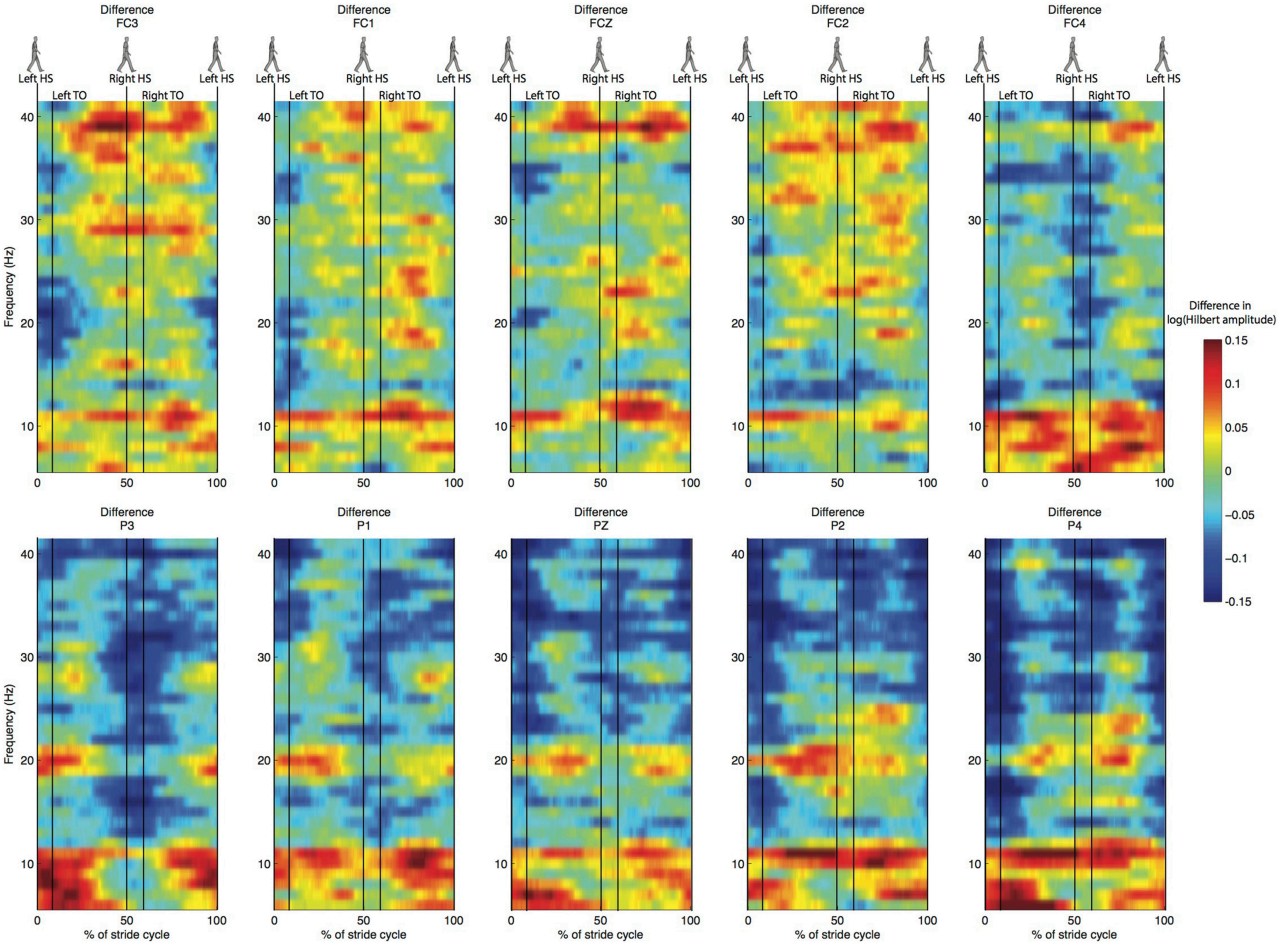
**Difference in normalized time-frequency analysis between normal and stabilized walking for the same electrodes as in Figures 4, 5, values below zero represent higher power in the stabilized condition**. Note that differences are not statistically significant.

Spectral analysis of the cleaned sensor data suggested differences between normal and stabilized walking in the lower beta band (around 18 Hz), as expected from previous studies (Wagner et al., 2012; Sipp et al., 2013). These differences, however, were not significant (Figure 4). Clear modulations in power were visible across the power spectrum (Figure 5). However, there were no significant differences between conditions (Figure 6).

#### Beamforming

**Figure 7:**
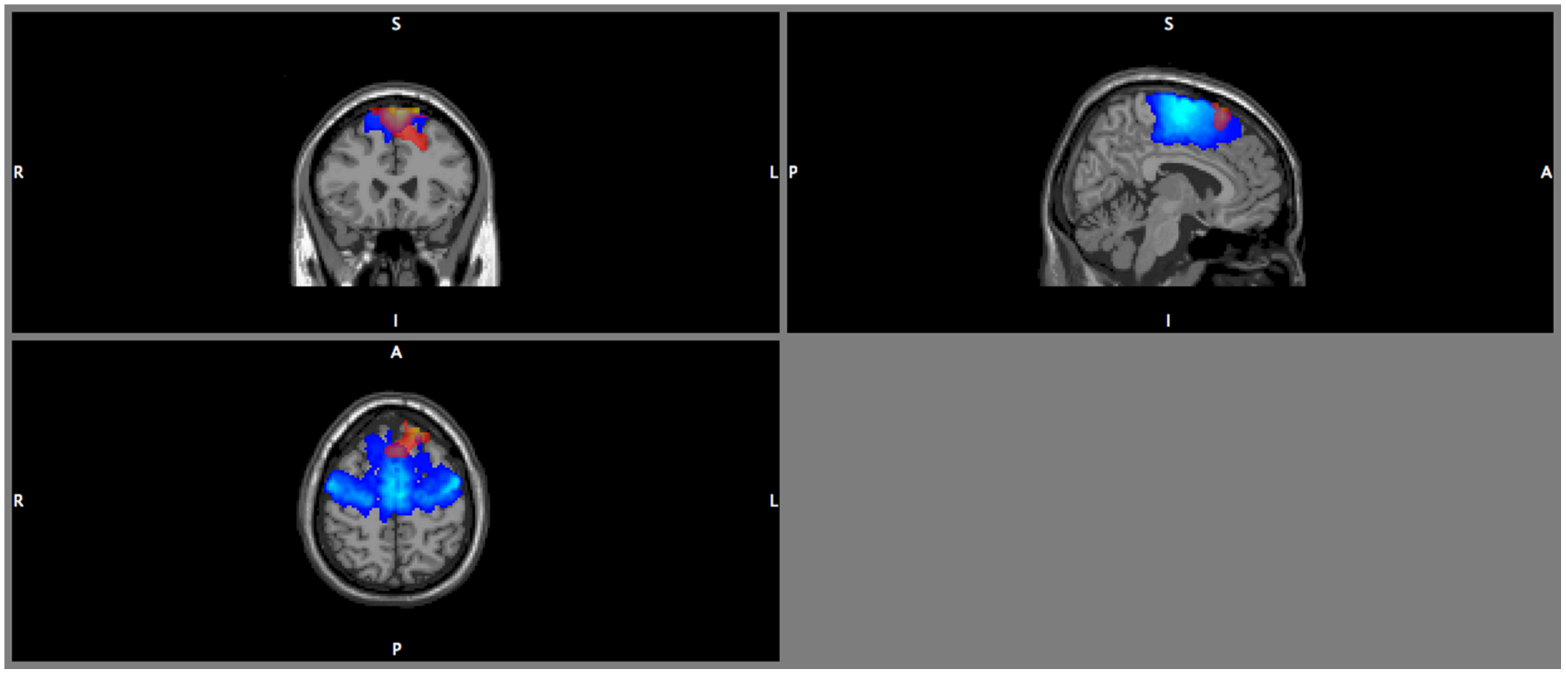
**DICS beamformer results for the contrast walking vs. sitting at 18 Hz**. Red color intensity indicates *t*-values at |t|> 4.5, blue color indicates the probability map for the left and right premotor areas. Note that the radiological convention was used (i.e., left is displayed right).

DICS beamforming yielded a cluster of significant *t*-values around bilateral pre-motor areas (Figure 7). Our criterion of separation of sources let to a total of two sources (Table 2), one in the left and one in the right premotor cortex. More sources were obtained but peak *t*-values dropped below 5 after the first two sources and mean *t*-values within a cluster with a radius of 2 cm around the peak dropped below 3 (Table 2 top row; Supplementary Figure 7).

**Table 2:** **Mean and individual locations (CTF coordinates) of sources for the contrast walking vs. sitting**.

	**Source 1: t_mean_ = −4.2; t_min_ = −6.3**			**Source 2: t_mean_ = −3.5; t_min_ = −5.3**		
	**X**	**Y**	**Z**	**X**	**Y**	**Z**
Average Subject	−17	35	61	28	15	66
1	−7	40	61	33	30	56
2	−2	35	51	43	15	56
3	−7	40	61	13	25	66
4	−27	35	46	13	5	71
5	−17	25	66	23	10	71
6	−12	45	46	28	15	66
7	−7	40	61	33	30	56
8	−2	30	51	23	30	56
9	−22	45	51	23	0	76
10	−22	45	51	23	5	71

*Mean sources were defined as the maximum t-value, and a sphere of 2 cm surrounding it, and distinct sources could not be overlapping. Displayed for means sources are location, peak t-value and mean t-value within the source. Individual sources were found within the volume of significant t-values. For each subject, the maximum power difference between normal walking and sitting conditions within this significant volume was determined*.

#### Source level analysis

**Figure 8:**
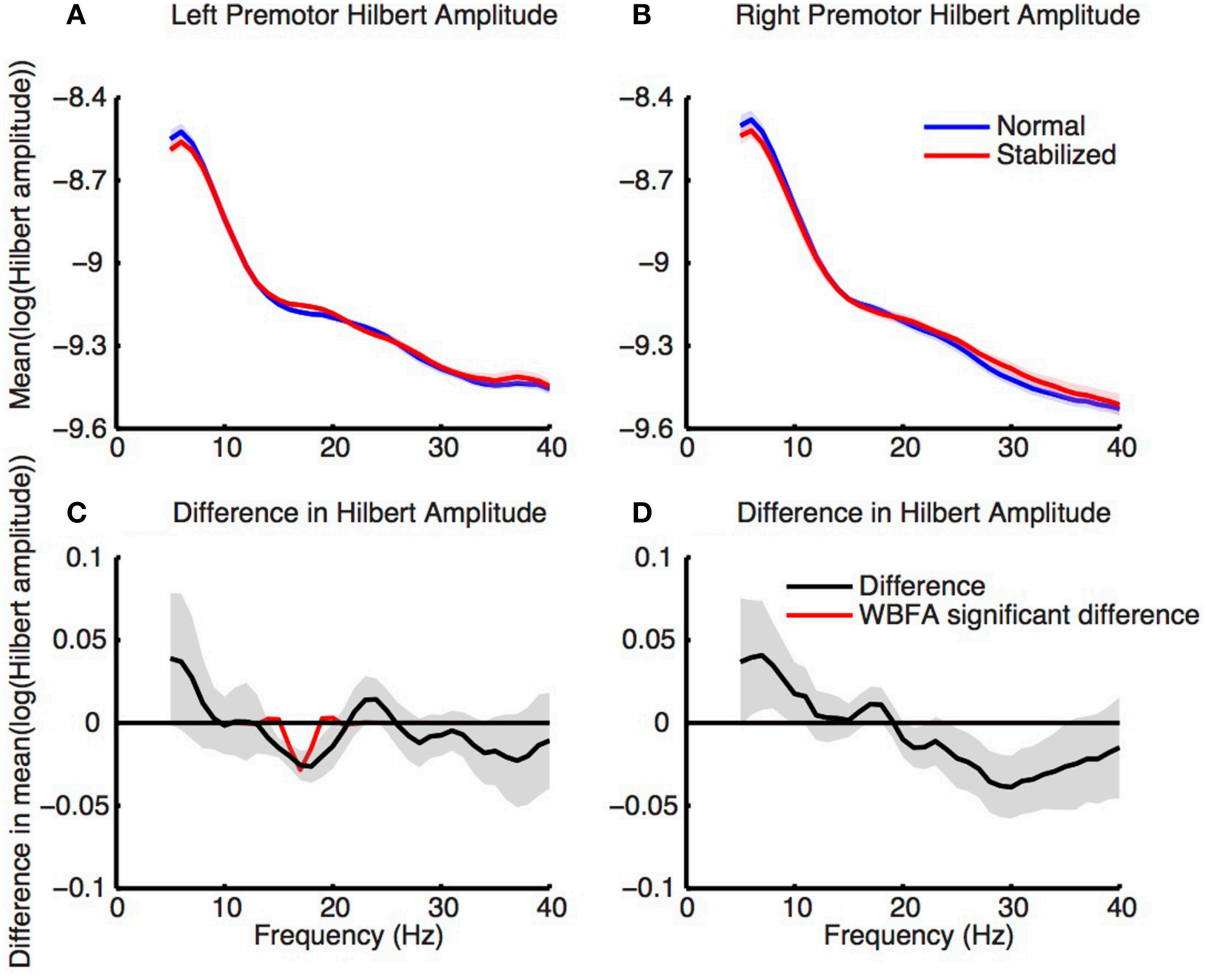
**Normalized power spectra (estimated via Hilbert transform) of (A) left and (B) right premotor areas during normal (blue) and stabilized (red) walking**. Lower panels **(C,D)** represent differences between conditions, with values below zero representing higher power in the stabilized condition. Red lines in the lower panels are the inverse wavelet transform of the significant wavelets, thus indicating the statistically significant differences in power between conditions. Shaded areas represent standard errors.

**Figure 9:**
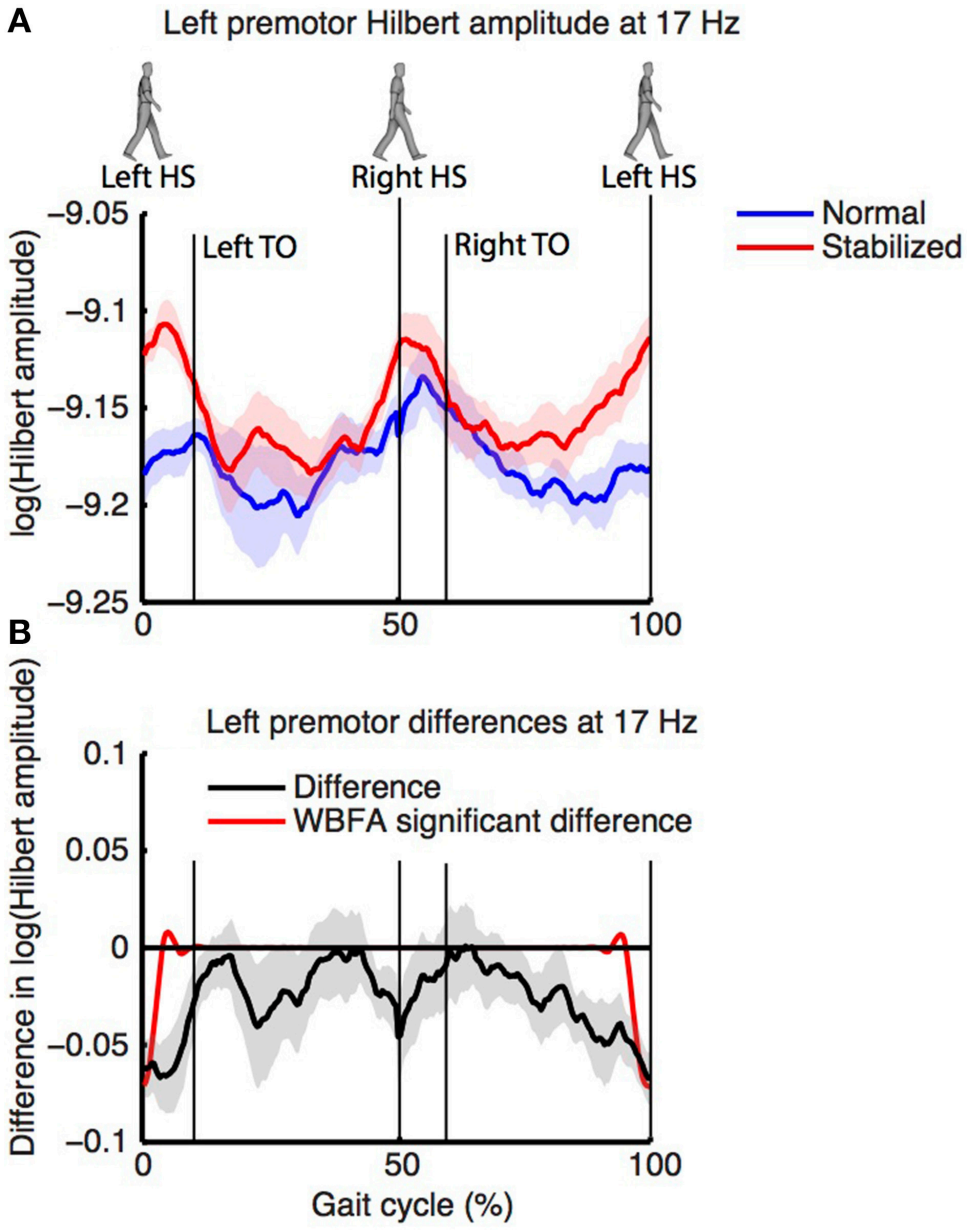
**(A)** Normalized power over the gait cycle (estimated via Hilbert transform) of left premotor area (at 17 Hz) during normal (blue) and stabilized (red) walking. Panel **(B)** displays the difference between conditions, with values below zero representing higher power in the stabilized condition. Shaded areas represent standard errors.

Figure 8 shows the log-transformed Hilbert amplitude-spectra derived from the signals projected on the left and right premotor areas. For the left premotor area, wavelet based functional ANOVA indicated increased amplitudes in the beta band (around 17 Hz) during stabilized walking. Considering the modulation of the Hilbert amplitude in this frequency band over the gait cycle (Figure 9, for modulation of non-significant right side, see Supplementary Figure 8), a depression in beta power was observed during single support (i.e., around 30 and 80% of the gait cycle) in both normal and stabilized walking. Significant differences in modulation between conditions were present only around left heel strike (i.e., at the end and beginning of the gait cycle), where beta power during stabilized walking was significantly higher. A similar increase in beta power in the stabilized condition with respect to the normal walking condition appeared to be present around right heel strike, but this effect was not significant.

## Discussion

We used a well-established stabilization paradigm (Donelan et al., 2004; Ijmker et al., 2013) to study how cortical areas are involved in controlling gait stability. Our behavioral results confirm previous studies. To our best knowledge, however, we are the first to show that also local divergence exponents decrease during stabilized walking. Thereby we show that not only trunk movements and step width decrease, but that stability does increase, as expected from the manipulation. As hypothesized, there was an increased beta activity during stabilized walking, and differences in modulations of beta power over the gait cycle between stabilized and normal walking.

Analyzing EEG during gait requires substantial data processing, and a recent study showed that EEG data recorded during gait that are not sufficiently cleaned contain spectral content that is similar to that of head acceleration (Castermans et al., 2014). We removed independent components with a frequency spectrum reminiscent of movement artifacts (Castermans et al., 2014; Kline et al., 2015) even if that drastically reduced the amount of independent components (although not as drastic as in some other studies, cf. Wagner et al., 2012; Sipp et al., 2013; Kline et al., 2014). Analysis of the SEP conditions showed that scalp maps of SEPs improved, with minor effects on amplitude, suggesting data cleaning was adequate, and not too aggressive. Still, we cannot prove that our results do not contain any movement artifacts. While several other studies (Gwin et al., 2011; Wagner et al., 2012; Sipp et al., 2013; Lau et al., 2014) used ICA not only for artifact rejection, but also as a source localization method (by fitting a dipole to a scalp map corresponding to an IC), we opted for more conventional beamformer methods. To the best of our knowledge, only a single study (Seeber et al., 2014) investigated EEG during gait in a similar way but employed sLORETA, with subject-specific MRIs. The advantage of using DICS beamformers over ICA dipole fitting is that it directly accounts for source activity in distinct frequency bands, in our case beta band activity.

The changes in SEP caused by both cleaning and condition were not significant. Interestingly, walking reduced the amplitude of the SEP, although not significantly so. This would suggest that sensory information from the wrist is suppressed, potentially because of the need to attend to other sensory information related to walking (Rushton et al., 1981). This, however, remains rather speculative because (1) this finding was non-significant, and (2) we did not randomize the order of walking and sitting SEP conditions, i.e., we cannot exclude potential ordering effects and/or structural changes in impedance due to drying electrode gel.

In general, motor control is accompanied by a depression in beta power (Pfurtscheller et al., 1996). Our results of an increased beta power during stabilized walking thus suggest decreased control. They are in line with the findings of Sipp et al. (2013), who reported increased beta power when walking normally as compared to walking on a balance beam. They also agree with the findings of Wagner et al. (2012), who reported an increased beta power in robot-assisted gait when compared to normal gait. Since the demands for propulsion and/or body weight support do not differ between walking with and without stabilization, it seems reasonable to assume that the increase in beta activity is directly related to a reduced demand to stabilize gait. However, a potential confounder may be the somatosensory input, which was different during the stabilization condition, as subjects wore an extra harness during the stabilized condition. Still, it is unlikely that this affected our results, as one would expect this to cause differences in the sensory cortex, rather than the premotor cortex.

We manipulated stability and observed changed in brain activity, thereby showing an association between the requirement to stabilize gait in ML direction and low beta activity in the premotor cortex. We believe that this does provide a proper indicator for—but not definitive proof of—the hypothesis that this brain activity reflects control of mediolateral stability by the premotor cortex. In order to establish the causality in the association, experimental manipulation of brain activity (for instance by means of transcranial direct current stimulation, tDCS), would be an obvious next step. While we are unaware of any studies performing tDCS during or prior to gait to influence gait stability, several groups have shown that postural control can be improved using tDCS (Sohn et al., 2013; Verheyden et al., 2013; Saeys et al., 2014).

We are not the first to report beta modulations during the gait cycle (Severens et al., 2012; Wagner et al., 2012; Seeber et al., 2014). We add to previous findings by reporting an overall increased beta power during stabilized walking, which suggest that beta power is related to gait stability. Variation in beta power over the gait cycle may be related to variation in the need for stabilizing control over the gait cycle. There is some evidence, indeed, that distinct phases in the gait cycle have a different stability. For example, when performing a simple reaction time task during gait, responses are slower during single support than during double support, suggestive of an increased “cognitive load” during single support (Lajoie et al., 1993). This is in line with our finding that in both our conditions beta power was lowest during single support, but cannot explain the differences in beta modulation between conditions, which occurred mostly during double support (i.e., ipsilateral heel strike, contralateral push-off).

Reducing the magnitude of the base of support (as during single support) may cause gait to be less stable (requiring more motor control) during single support. However, one should not forget that gait is dynamic rather than static, and that during single support the center of mass is not within the base of support, nor should it be. Why may single support require extra motor control? One likely option is that during single support, the placement of the swing foot is planned. Several studies have shown that either trunk or body center of mass state during mid-stance predicts foot placement in the subsequent step (Hurt et al., 2010; Rankin et al., 2014; Wang and Srinivasan, 2014). During single support, information about the state of the body probably needs to be integrated, to form a motor command to place the swing leg in the right position to maintain stability.

Although we did find a lower beta power during single support, the major differences between conditions occurred during double support, the phase in which contralateral push-off occurs. Here, stabilized walking showed a significantly higher beta power, implying less motor control. This would be in line with the findings of Ihlen et al. (2012) who reported that gait is unstable during weight transfer. Moreover, in a recent study, Kim and Collins (2015) showed that a “once-per-step” control of an ankle-foot prosthesis during push-off, reduced the effort associated with medio-lateral stability control during walking, and similar findings have been reported in model studies (Kim and Collins, 2013; Fu et al., 2014). All in all, these findings suggest an important role for a well-controlled push-off to maintain a stable gait pattern, and our findings suggest that there may be cortical involvement in such control.

Although beamforming initially resulted in sources in left and right premotor areas, subsequent analysis of the data as projected on these sources only showed significant differences for the left premotor area. Recent studies also suggest an important role for the left hemisphere. For instance, Bruijn et al. (2014) found that white matter microstructural integrity in left cortico-spinal tract and anterior thalamic radiation was correlated to measures of foot placement, and Sipp et al. (2013) showed that the left sensorimotor cortex was the first area to respond to a loss of balance while walking on a balance beam. These findings seem to converge on a specialized role for the left hemisphere in the control in gait stability. As of yet, however, supporting evidence from lesion based studies (for instance after stroke) is largely absent, and it might be interesting to further investigate whether patients who suffered a stroke in the left hemisphere suffer more from stability problems than those who suffered a stroke in the right hemisphere.

In conclusion, during stabilization of gait by elastic bands, we found increased beta band activity in the left premotor cortex, suggesting that this area is involved in maintaining steady state gait stability. The modulation of this activity appears in line with the idea that medio-lateral foot placement is at least in part determined during push-off, although more work is needed to confirm this hypothesis.

### Conflict of interest statement

The authors declare that the research was conducted in the absence of any commercial or financial relationships that could be construed as a potential conflict of interest.

## Footnotes

^1^Strictly speaking this definition does not provide a “power” as we used the modulo of the band-pass filtered analytic signal, i.e., the time and frequency dependent Hilbert amplitude without squaring it.

^2^We also performed this procedure for walking vs. stabilized walking, but this yielded invalid results (see Supplementary Figure 1), most likely due to the small differences between these conditions, combined with the high noise levels. We also performed the procedure for stabilized walking vs. sitting, which yielded similar results as walking vs. sitting see Supplementary Figure 2, which made us confident that the sources we found are active during both walking and stabilized walking.

^3^We also performed source localization of this evoked response using a multiple signal classification procedure. Results for this can be found in Supplementary Figures 3–6, and are similar to the results shown here, the cleaned data had more focal sources.

## Supplementary material

Supplementary Figure 1 **DICS beamformer results for the contrast walking vs. stabilized walking at 18 Hz**. Color intensity indicates *t*-values.

Supplementary Figure 2 **DICS beamformer results for the contrast stabilized walking vs. sitting at 18 Hz**. Color intensity indicates *t*-values. Figure is tresholded at |t|> 4.5.

Supplementary Figure 3 **Source localization (MUSIC) results for the SEP condition during sitting, without cleaning**.

Supplementary Figure 4 **Source localization (MUSIC) results for the SEP condition during walking, without cleaning**.

Supplementary Figure 5 **Source localization (MUSIC) results for the SEP condition during sitting, after cleaning**.

Supplementary Figure 6 **Source localization (MUSIC) results for the SEP condition during walking, after cleaning**.

Supplementary Figure 7 **Individual sources**. The green and blue circle represent the group peak *t*-values, the stars represent the individual subject sources used for analysis.

Supplementary Figure 8 **Figure 9 with results for right premotor area at 17 Hz also plotted**.
