# Supplementary figures and images for: Beta activity in the premotor cortex is increased during stabilized as compared to normal walking
Source: Front Hum Neurosci. 2015 Oct 27;9:593. doi: 10.3389/fnhum.2015.00593 (PMC4621867; doi:10.3389/fnhum.2015.00593)

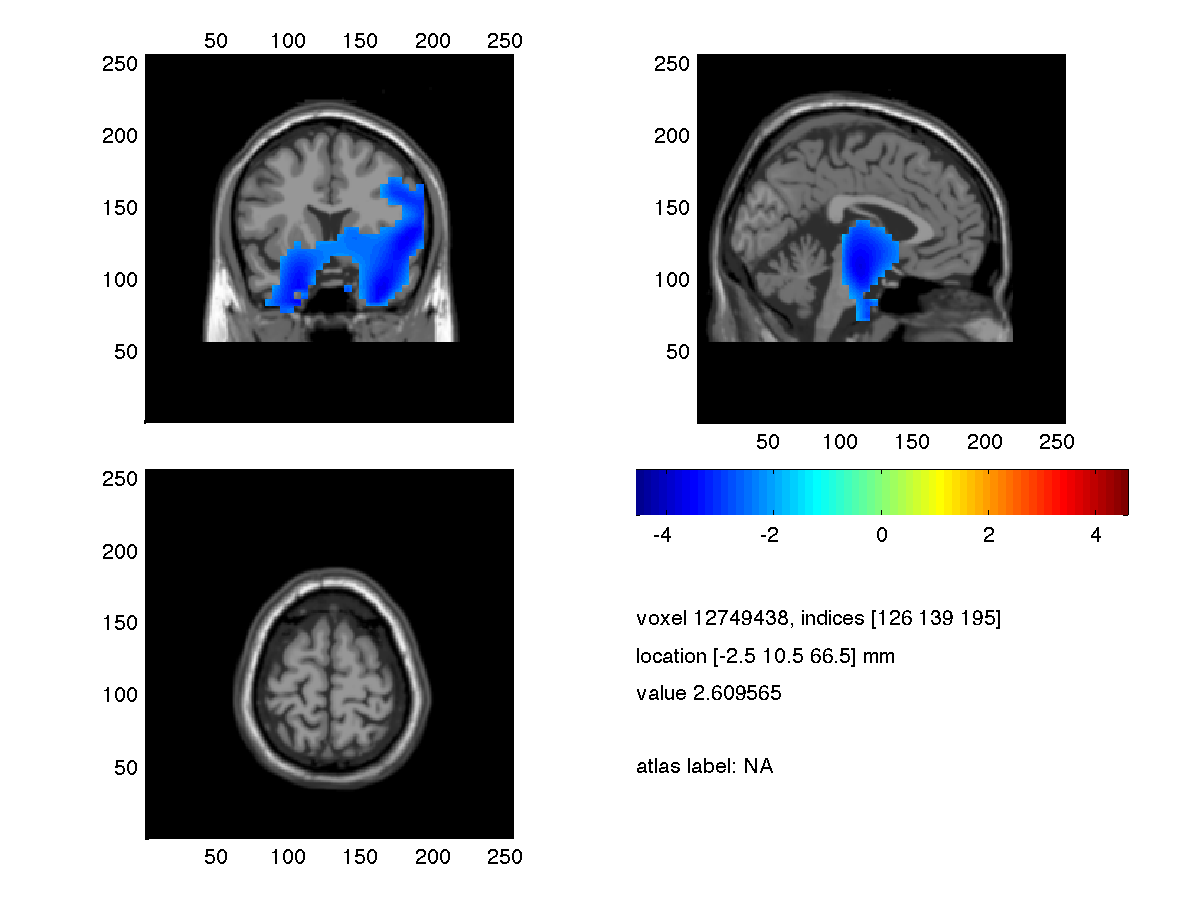

Supplement: Supplementary Figure 1 — DICS beamformer results for the contrast walking vs. stabilized walking at 18 Hz. Color intensity indicates t-values. [file Image1.TIFF]

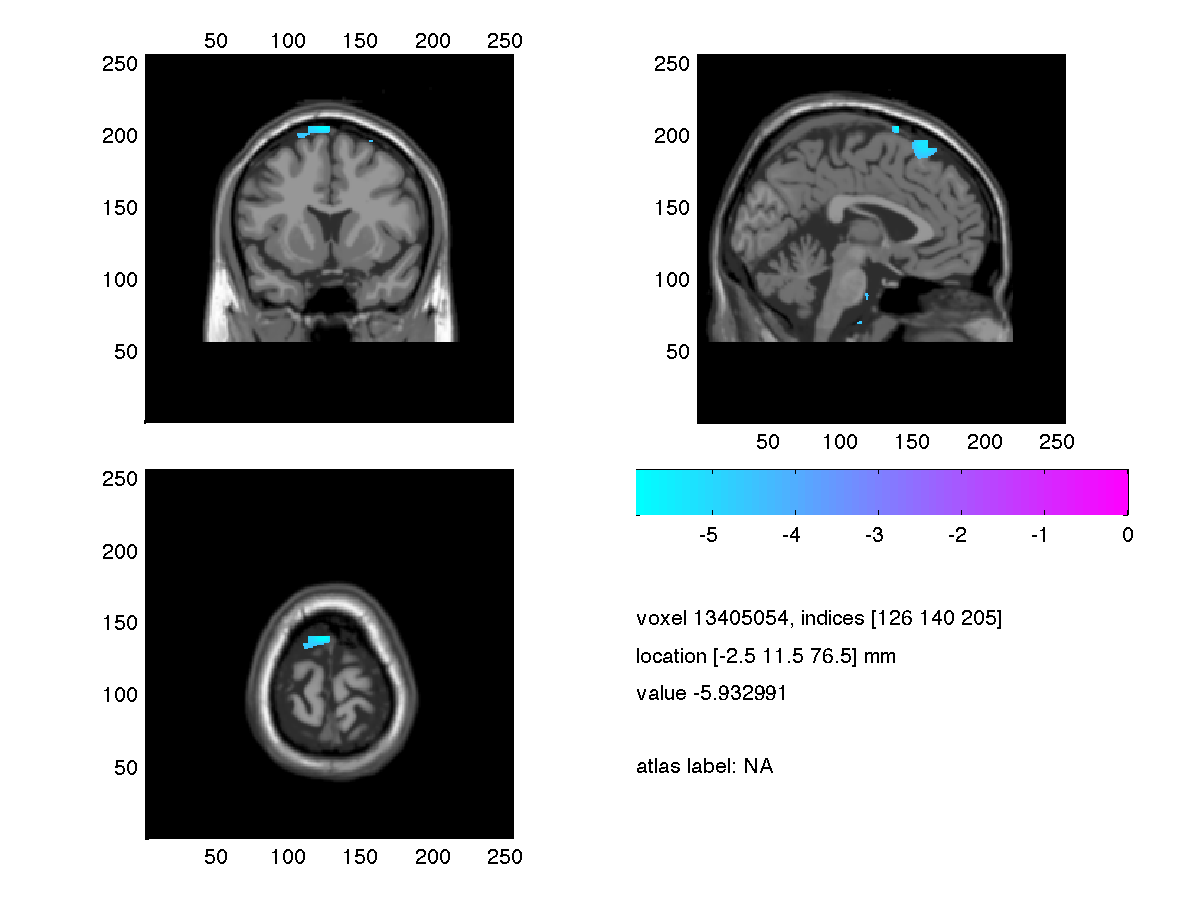

Supplement: Supplementary Figure 2 — DICS beamformer results for the contrast stabilized walking vs. sitting at 18 Hz. Color intensity indicates t-values. Figure is tresholded at |t|> 4.5. [file Image2.TIFF]

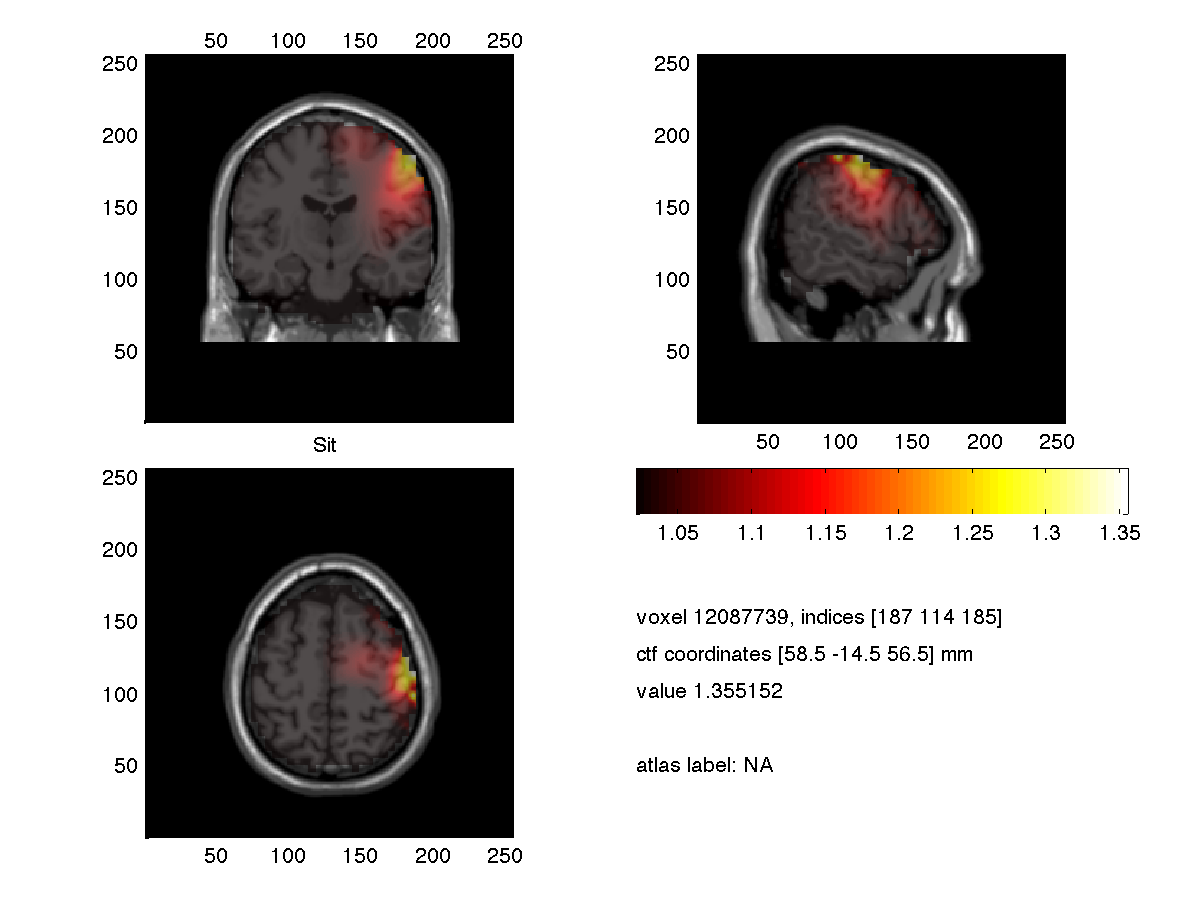

Supplement: Supplementary Figure 3 — Source localization (MUSIC) results for the SEP condition during sitting, without cleaning. [file Image3.TIFF]

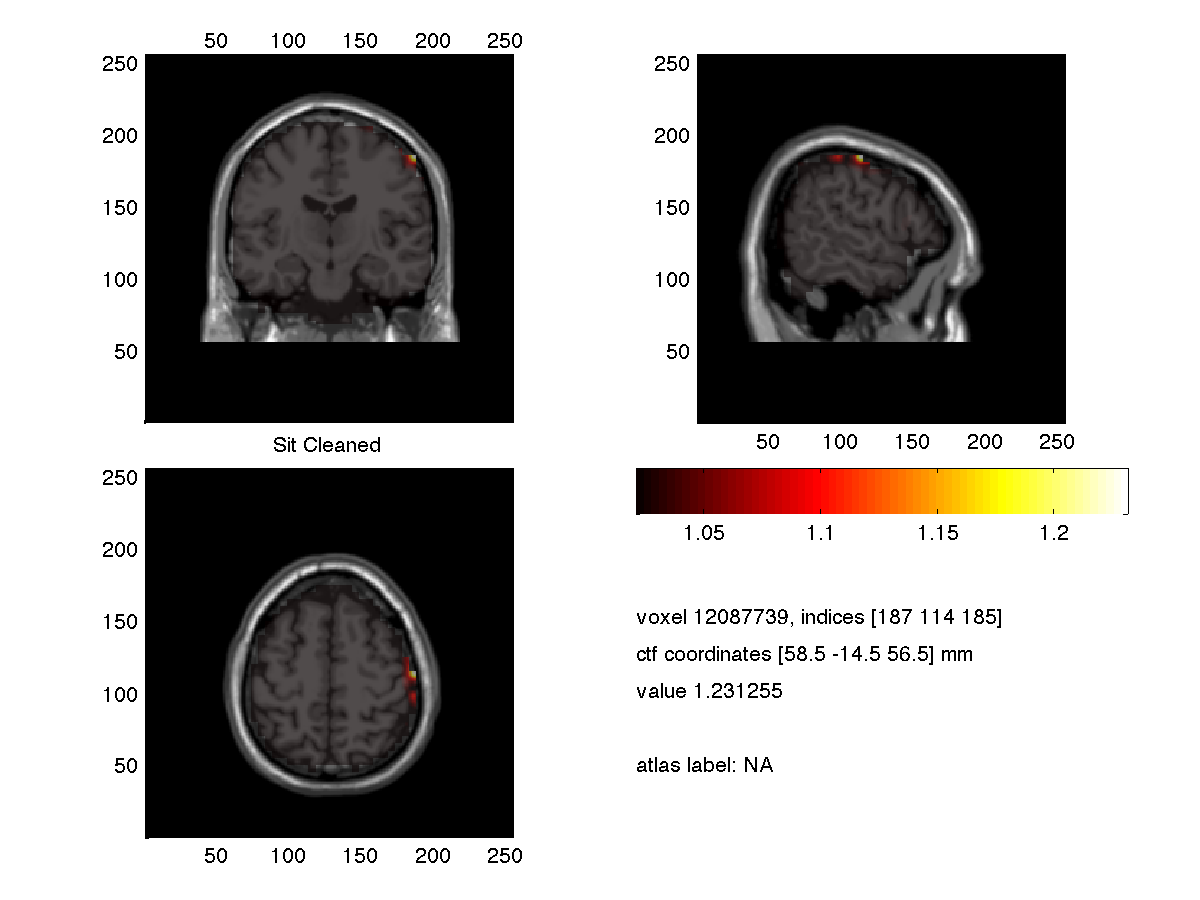

Supplement: Supplementary Figure 4 — Source localization (MUSIC) results for the SEP condition during walking, without cleaning. [file Image4.TIFF]

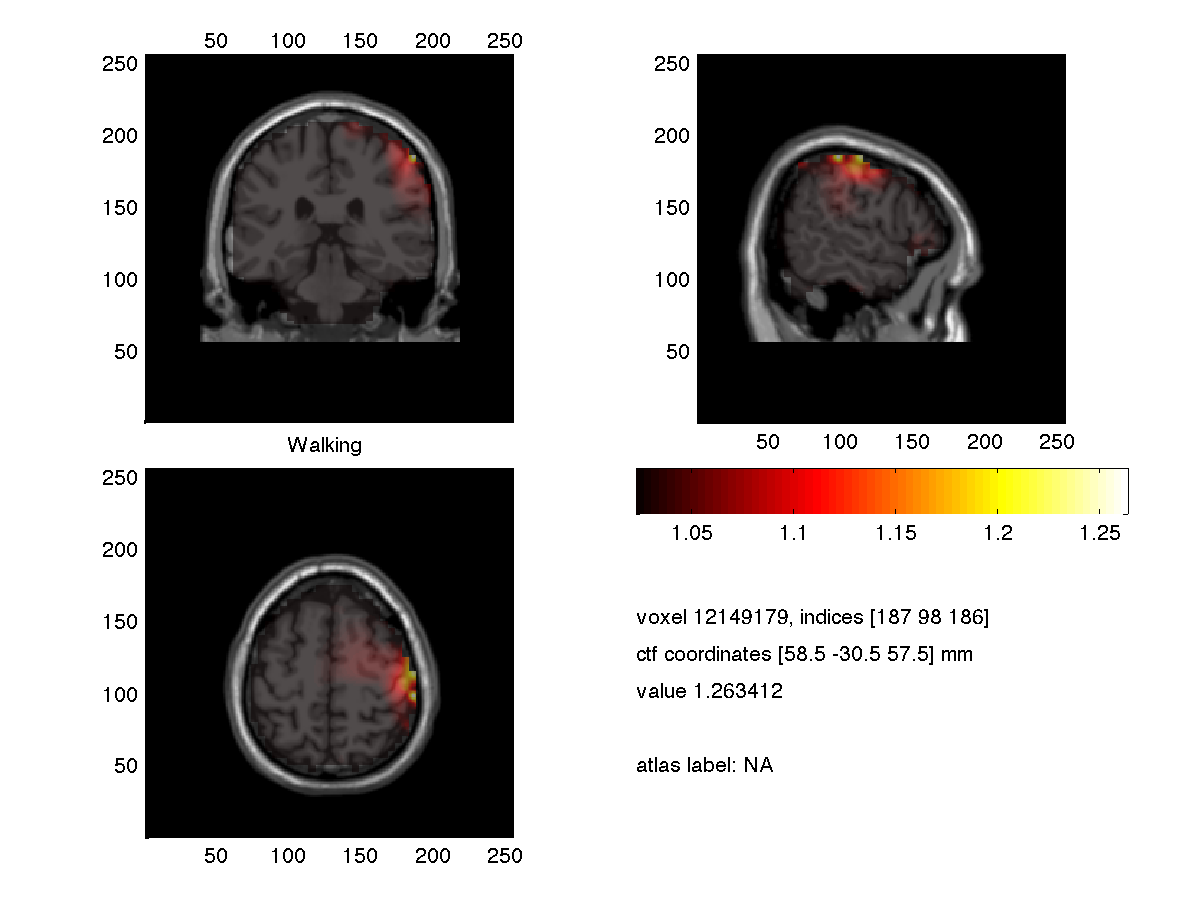

Supplement: Supplementary Figure 5 — Source localization (MUSIC) results for the SEP condition during sitting, after cleaning. [file Image5.TIFF]

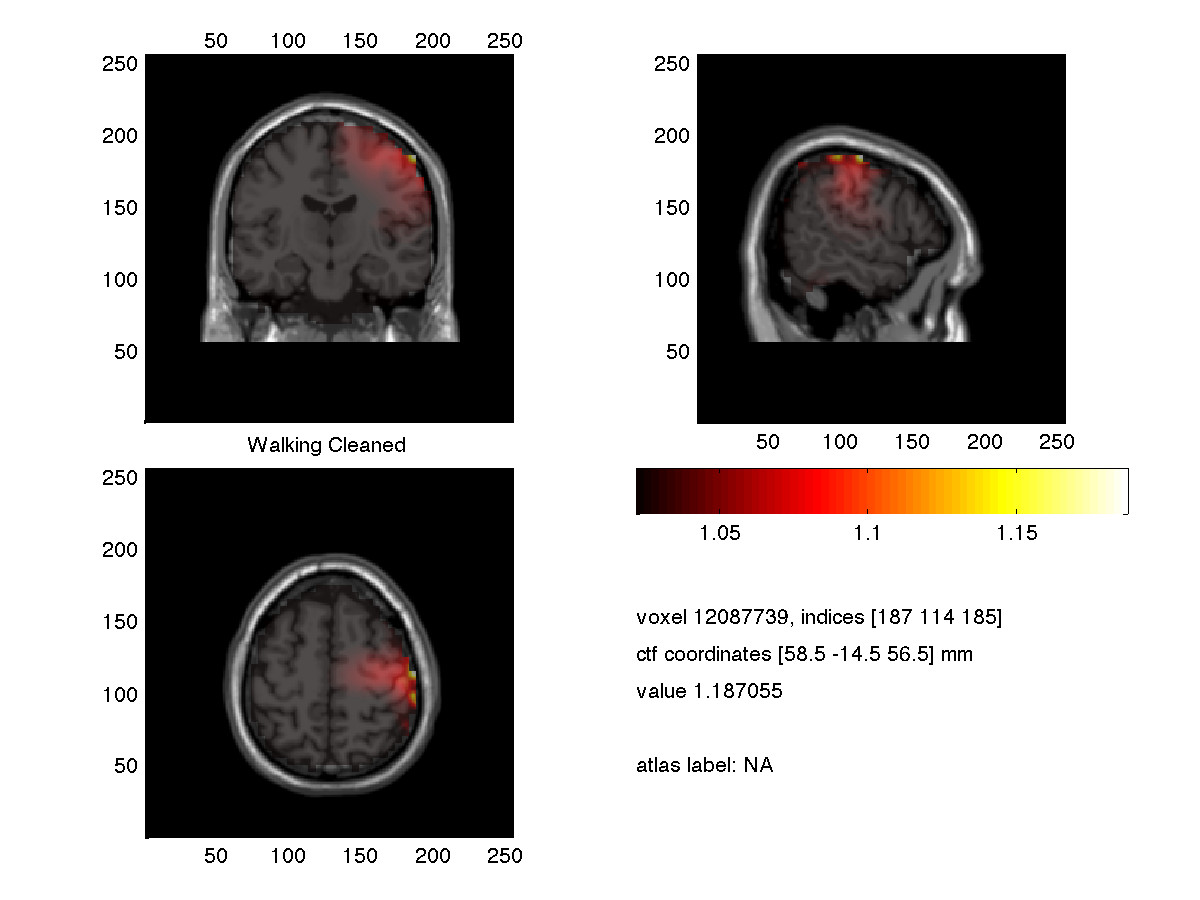

Supplement: Supplementary Figure 6 — Source localization (MUSIC) results for the SEP condition during walking, after cleaning. [file Image6.TIFF]

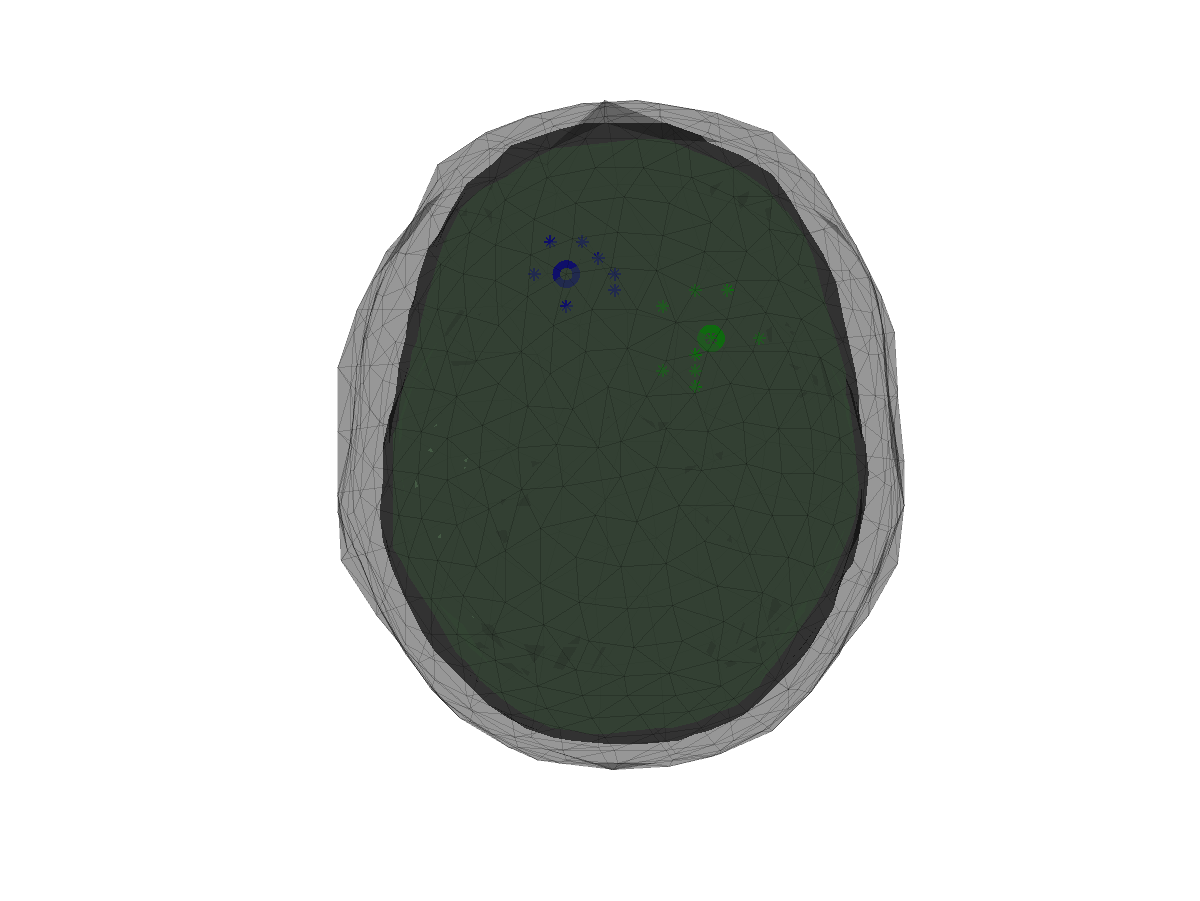

Supplement: Supplementary Figure 7 — Individual sources. The green and blue circle represent the group peak t-values, the stars represent the individual subject sources used for analysis. [file Image7.TIFF]

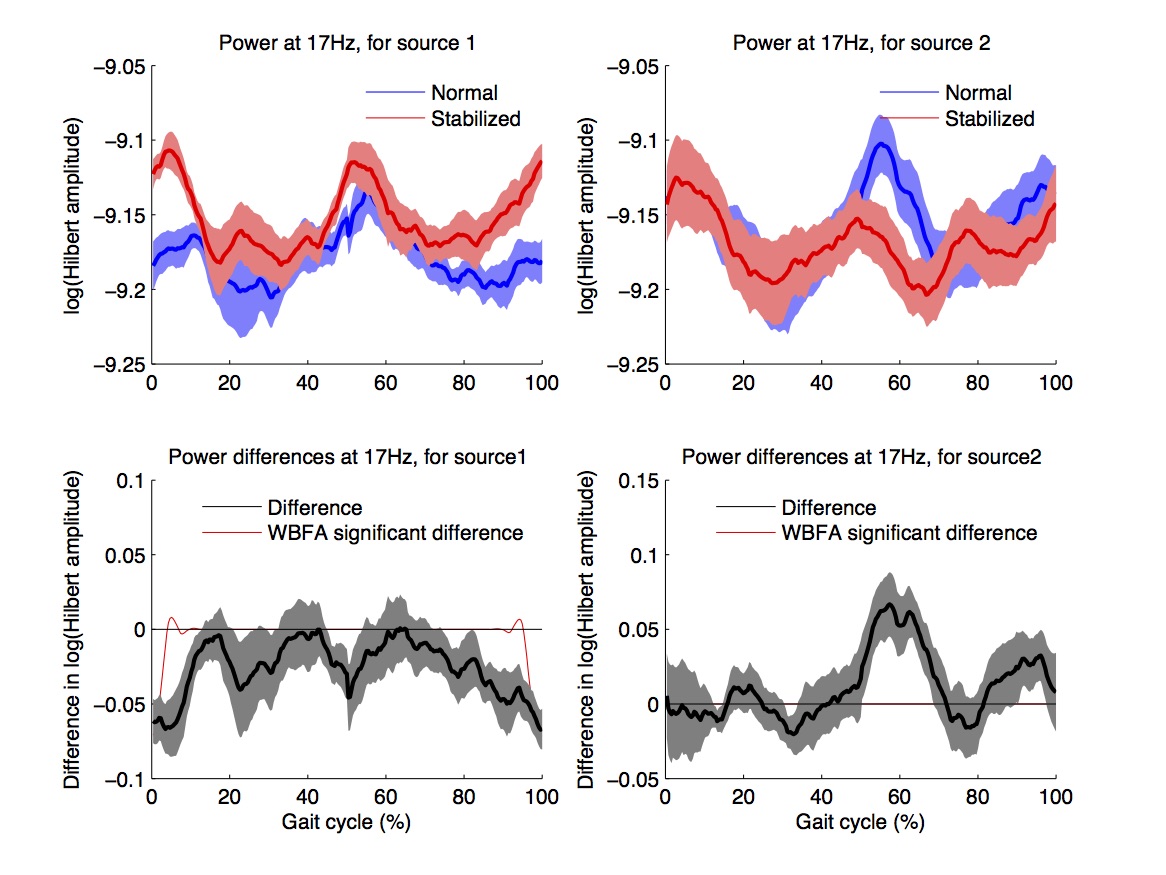

Supplement: Supplementary Figure 8 — Figure 9 with results for right premotor area at 17 Hz also plotted. [file Image8.JPEG]
